# Evaluating the association between extreme heat and mortality in urban Southwestern Ontario using different temperature data sources

**DOI:** 10.1038/s41598-021-87203-0

**Published:** 2021-04-14

**Authors:** Kristin K. Clemens, Alexandra M. Ouédraogo, Lihua Li, James A. Voogt, Jason Gilliland, E. Scott Krayenhoff, Sylvie Leroyer, Salimah Z. Shariff

**Affiliations:** 1 ICES, Toronto, ON Canada; 2 Department of Medicine, Western University, London, ON Canada; 3 Department of Epidemiology, Western University, London, ON Canada; 4 Lawson Health Research Institute, London, ON Canada; 5 Department of Geography, Western University, London, ON Canada; 6 Department of Pediatrics, Western University, London, ON Canada; 7 School of Health Studies, Western University, London, ON Canada; 8 School of Environmental Sciences, University of Guelph, Guelph, ON Canada; 9 Meteorological Research Division, Environment and Climate Change Canada, Gatineau, Canada; 10 St. Joseph’s Health Care London, 268 Grosvenor Street, London, ON N6A 4V2 Canada

**Keywords:** Environmental sciences, Medical research

## Abstract

Urban areas have complex thermal distribution. We examined the association between extreme temperature and mortality in urban Ontario, using two temperature data sources: high-resolution and weather station data. We used distributed lag non-linear Poisson models to examine census division-specific temperature–mortality associations between May and September 2005–2012. We used random-effect multivariate meta-analysis to pool results, adjusted for air pollution and temporal trends, and presented risks at the 99th percentile compared to minimum mortality temperature. As additional analyses, we varied knots, examined associations using different temperature metrics (humidex and minimum temperature), and explored relationships using different referent values (most frequent temperature, 75th percentile of temperature distribution). Weather stations yielded lower temperatures across study months. U-shaped associations between temperature and mortality were observed using both high-resolution and weather station data. Temperature–mortality relationships were not statistically significant; however, weather stations yielded estimates with wider confidence intervals. Similar findings were noted in additional analyses. In urban environmental health studies, high-resolution temperature data is ideal where station observations do not fully capture population exposure or where the magnitude of exposure at a local level is important. If focused upon temperature–mortality associations using time series, either source produces similar temperature–mortality relationships.

## Introduction

With rising global temperatures, there has been increased attention upon the association between ambient temperature, morbidity, and mortality. Extremes in temperature have been linked with cardiovascular and respiratory events, heat illness, and even death^1–3^.

The health impact of extreme temperature on urban communities warrants special attention. Cities not only have high population density and house heat-vulnerable communities (e.g. older adults, homeless)^4^, but they have a complex microclimate. Thermally, cities exhibit higher surface and near-surface atmospheric temperature than non-urbanized areas due to modifications to the cover, form and materials used in these regions (i.e. urban heat island effect). During heat waves governed by large-scale meteorological conditions, urban heat island effects can also amplify or prolong the duration of heat events^5^.

When conducting environmental health studies in cites, it is important to use the right temperature data source. Most studies use weather stations^1,2,6–8^, but if situated near airports, water or grassy areas, stations might not adequately capture complex thermal distribution in cities, particularly where stations are sparse and remote^9,10^. In cities that incorporate irrigation that sustains more vegetation, and in those with shading effects between buildings, there might also be daytime temperature bias when rural weather stations are used.

Outside of Canada, an increasing number of studies have used high-resolution temperature data to capture complex temperature exposure in urban regions^11–15^. In our country, weather stations are still most often used^2,8,16^. Given ambient temperatures in Canada are increasing well beyond global rates^17^, it is important to have a full understanding of the benefits and limitations of temperature data sources available for urban heat-health studies.

With access to rich administrative health data and a contemporary high-resolution weather data source (Canadian Urban and Land Surface Modeling System, or GEM-SURF), we examined the relationship between temperature and mortality in urban Southwestern Ontario using two data sources available to researchers: GEM-SURF and weather stations.

## Methods

### Design and setting

We conducted population-based time series analyses to investigate the association between temperature and mortality in urban Ontario, Canada. Given heat-health risks vary regionally^14^, we focused upon Southwestern Ontario^18^, a 21,639 km^2^ region, bounded North to South by the Bruce Peninsula and Lake Erie (latitudes 41.92–45.23), and West to East by Windsor and Niagara Falls (longitudes − 83.11 to − 78.93). Southwestern Ontario has moderate humid continental climate, with warm to hot humid summers^19^.

In Ontario, people have universal access to hospital, diagnostic, and physician services. Information on use of health services is collected and maintained in databases held at ICES (formerly the Institute for Clinical Evaluative Sciences). Datasets are linked using unique encoded identifiers and analyzed at ICES. We followed the REporting of studies Conducted using Observational Routinely-collected Data Statement (Supplementary Table S1)^20^.

### Population

We included all residents of urban Southwestern Ontario who died of a non-accidental cause between May and September 2005–2012. We defined urban areas as regions with ≥ 10,000 residents^21^. We focused upon May to September as temperatures are highest during these calendar months in our region^22^, we expected temporal variation in temperatures over this period, and we anticipated that higher temperatures earlier (May) and later (September) in the season might be unexpected and more dangerous^12^.

We excluded residents: (1) with missing or invalid identification numbers that precluded linkage; (2) who were not Ontario residents; and (3) who lived in long-term care as these individuals might have different risks of temperature-related outcomes^23^. We also restricted to individuals who lived in a region where a temperature exposure could not be assigned using our data sources (described below).

### Data sources

Data sources included health administrative and environmental weather data. ICES health administrative databases included the Registered Persons Database of Ontario which was used to ascertain demographic information. This database contains information for all those who received a health card in our province. We obtained causes of death from the Office of the Registrar General Death Database. We used the Ontario Local Health Integration Network administrative boundaries (i.e. regions 1–4) to determine the geographic location of Southwestern Ontario residents (Supplementary Fig. S1).

We captured characteristics and comorbidities using databases including the Ontario Marginalization Index, a geographically-based index that quantifies degrees of marginalization (residential instability, material deprivation, dependency, and ethnic concentration)^24^. We used the Canadian Institute for Health Information’s Discharge Abstract Database and the National Ambulatory Care Reporting System Database to assess comorbidities. These databases contain diagnostic and procedural information gathered during inpatient hospital stays and emergency department encounters respectively (via International Classification of Diseases [ICD] and Canadian Classification of Health Interventions codes). We further used datasets derived from validated case definitions of comorbidities, including the Congestive Heart Failure^25^, Chronic Obstructive Pulmonary Disease^26^, Ontario Diabetes^27^, and Hypertension datasets^28^. The Ontario Health Insurance Plan database contains physician diagnostic and billing information, and was used for additional covariates. A list of the administrative codes used in this study is included in Supplementary Table S2.

We acquired weather station and high-resolution estimates of daily temperature from Environment and Climate Change Canada (ECCC). GEM-SURF (high-resolution, urbanized data source) was developed to predict weather and air quality in densely populated urban regions, and provides hourly high-resolution (1 km × 1 km) hindcasts of surface and near-surface meteorological variables in urban environments of various densities. GEM-SURF validation studies have examined energy balance fluxes and radiative surface temperature, and ground-based validations using vehicle traverse or tower sites have been completed across multiple cities, seasons, and types of topography in Canada^29–33^. In Montreal Quebec, air temperature bias was − 1/+ 1 K^32,33^. In Toronto Ontario and Vancouver British Columbia, air temperature bias was < 0.5 degree Celsius (°C) with a standard deviation error < 1.3 °C^29,31^. GEM-SURF accurately identifies intra-urban hotspots, and has improved model performance when urban surfaces are included^29–33^.

To facilitate linkage of GEM-SURF with ICES data sources, we aggregated temperature to the daily and dissemination area [DA] level (small geographic areas composed of one or more neighborhoods, population of 400–700 persons)^34^. Consistent with previous studies, we assigned temperature exposure based upon residential location^8^.

**Figure 1:**
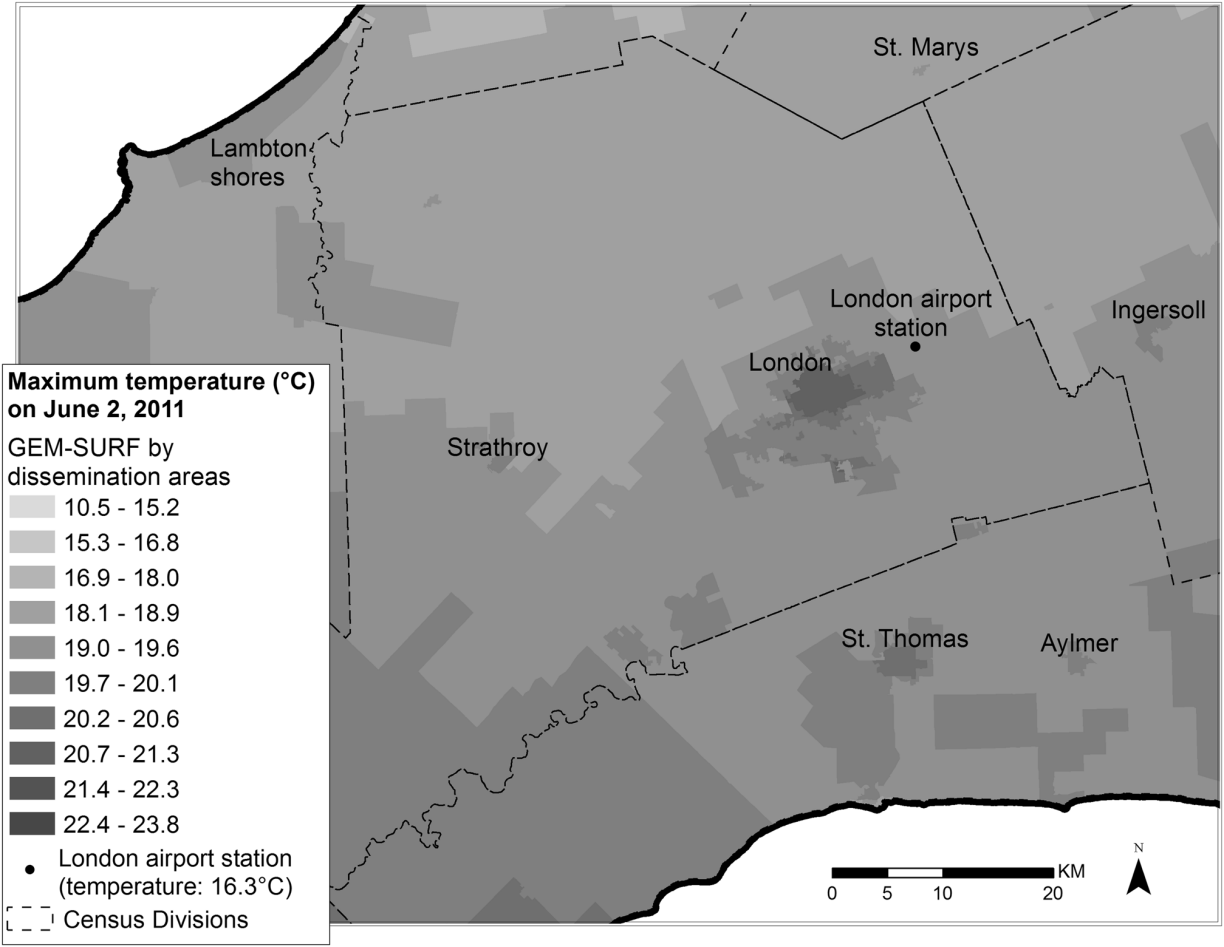
Comparison between maximum daily temperature ascertained by a single weather station on June 2nd 2011 (16.3 °C at London Ontario International Airport) and GEM-SURF temperatures across regional dissemination areas (10.5 to 23.8 °C). Map created by A.O. using ArcGIS software version 10.3 by ESRI, www.esri.com.

As our second temperature data source, we used hourly data from weather stations (Supplementary Fig. S1)^35^. ECCC weather station data has < 1 °C measurement bias, consistent with international standards (decimal precision of 0.1 °C)^36^. Due to the scarcity of stations in some areas, we summarized daily temperature values to the census division level (i.e. county or regional district)^21^. If only a single weather station was located within a particular census division, we used daily station values to estimate exposure for the entire census division. If there were multiple monitoring stations in a census division, we calculated the average of measures to produce an overall exposure estimate. Figure 1 illustrates differences in temperature exposure with use of GEM-SURF vs. weather stations. The maximum daily temperature from a single weather station on a single day is provided along with nearby temperatures from GEM-SURF, summarized by DA. The weather station, located outside the urban core at the London Ontario Airport reported a temperature of 16.3 °C. The temperature assigned by GEM-SURF in the urban core ranged between 18 and 21.3 °C.

Finally, we obtained air quality data from Ontario Ministry of the Environment monitoring stations over the study period. We included nitrogen dioxide (NO_2_), ozone (O_3_) and fine particulate matter (< 2.5 µm in aerodynamic diameter) (PM_2.5_)^37^. We derived mean daily pollution estimates for each census division.

### Main exposure

While there is debate as to the best temperature variable to use in temperature-morbidity/mortality studies, we chose maximum daily temperature as our main exposure, given its best representation of daytime temperature^7,38^.

### Outcomes

Our primary outcome was non-accidental death (daily count) between May and September 2005–2012. We identified non-accidental causes of death using ICD-10th revision codes A00-R99.

### Analysis

We used descriptive statistics (e.g. means, standard deviations or SD, medians, interquartile ranges, numbers, percentages) to summarize the characteristics of those who died over the study period. Using both temperature data sources, we then fit distributed lag non-linear models (DLNM) with quasi-Poisson distribution to determine census division-specific associations between maximum daily temperature and mortality. DLNMs allow for the modelling of complex non-linear and lagged dependences in exposure-response relationships (e.g. temporal change in risk after an exposure)^39^. We estimated temperature–mortality associations using natural cubic splines with three internal knots at the 10th, 50th, and 90th percentiles of location-specific temperature distribution at a lag of 0–3 days. Based upon previous recommendations and our own data exploration, we allowed 7 degrees of freedom (df) per year to describe long-time trends and seasonality. Consistent with other studies, we also included an indicator variable for day of week, public holidays, and physician visits for influenza^2,6^.

Thereafter, we used random-effects multivariate meta-analysis to pool census division-level estimates and produce a summary level estimate for urban Southwestern Ontario. We tested for residual heterogeneity using the multivariate extension of the Cochran Q test and I^2^ statistics^39^. For our main analysis, we present pooled cumulative curves of the relationship between temperature and mortality, centered around the minimum mortality temperature (MMT, temperature at which mortality is at a minimum)^39^. We also present curves by census division.

As prespecified additional analyses, we changed the number and placement of knots for temperature using 4, 5, and 6 df and 3 and 4 df for lags. To evaluate for the confounding effect of air pollution^40^, we adjusted for linear terms of mean PM_2.5_, O_3_ and NO_2_ at a lag of 0–1 in our models. We also examined the relationship between mortality and humidex (a unitless index that indicates how hot and humid the weather feels to the average person)^41,42^.

We conducted a number of post-hoc analyses. We examined mortality risk per 1 °C increase in temperature at extreme hot temperatures, defined using the 99th percentile of weather station maximum temperature distribution, and used MMT as our referent. Recognizing that minimum daily temperature also shows urban heat effects^43^, we examined mortality risk per 1 °C increase in minimum temperature defined using the 99th percentile compared to the MMT. Further, to confirm the robustness of selecting MMT as our referent value, we examined temperature–mortality associations using the 75th percentile of weather station temperature distribution as the referent. Moreover, we explored the correlation between MMT and local most frequent temperature [MFT]^44^, and investigated temperature–mortality associations using MFT as the referent.

We present relative risks (RR) (i.e. risk of death at extreme temperature relative to the risk at a referent temperature) and 95% confidence intervals (CI). Estimates were considered statistically significant at p-values < 0.05. Analyses were performed using SAS version 9.4 [SAS Institute, Cary, NC] and R version 3.1.2.

### Ethical considerations

Use of ICES data in this study was authorized under section 45 of Ontario’s Personal Health Information Protection Act, which does not require review by a Research Ethics Board.

## Results

A flow diagram of inclusions and exclusions is provided in Supplementary Fig. S2. From May to September 2005–2012, there were 54,399 people with a non-accidental death. The characteristics of residents who died over the study period are presented in Table 1. The mean age of individuals at the time of death was 73 years, 46% were female, and 25% were from the lowest income quintile.

**Table 1:** Baseline characteristics for people who died in Southwestern Ontario between 2005 and 2012.

Characteristics	May–Sept
	N = 54,399
**Age (mean ± SD)**	73.03 ± 15.60
(median, IQR)	76 (65–84)
0–65 years	14,333 (26.3%)
66+ years	40,066 (73.7%)
Female	25,189 (46.3%)
**LHIN**	
01	10,407 (19.1%)
02	10,723 (19.7%)
03	8730 (16.0%)
04	24,539 (45.1%)
**Neighborhood income quintile**	
Q1—lowest	13,411 (24.7%)
Q2	12,427 (22.8%)
Q3	10,636 (19.6%)
Q4	9114 (16.8%)
Q5—highest	8550 (15.7%)
Missing	261 (0.5%)
**Marginalization index—deprivation**	
1**—**least deprived	9236 (17.0%)
2	9287 (17.1%)
3	9731 (17.9%)
4	10,749 (19.8%)
5**—**most deprived	14,922 (27.4%)
Missing	474 (0.9%)
**Marginalization index—ethnic concentration**	
1—least diverse	12,376 (22.8%)
2	15,157 (27.9%)
3	13,460 (24.7%)
4	9487 (17.4%)
5—most diverse	3445 (6.3%)
Missing	474 (0.9%)
**Marginalization index—dependency**	
1—least dependent	4982 (9.2%)
2	7988 (14.7%)
3	9802 (18.0%)
4	11,856 (21.8%)
5—most dependent	19,297 (35.5%)
Missing	474 (0.9%)
**Marginalization index—instability**	
1—lowest instability	4601 (8.5%)
2	8080 (14.9%)
3	10,058 (18.5%)
4	12,721 (23.4%)
5—highest instability	18,465 (33.9%)
Missing	474 (0.9%)
CHF	17,828 (32.8%)
COPD	20,943 (38.5%)
Hypertension	38,320 (70.4%)
Diabetes	17,455 (32.1%)
Coronary artery disease (excluding angina)	21,525 (39.6%)
Cerebrovascular disease	7151 (13.1%)
Dementia	7926 (14.6%)
Chronic kidney disease	14,415 (26.5%)

*CHF* congestive heart failure, *COPD* chronic obstructive pulmonary disease, *LHIN* Local Health Integration Network, *SD* standard deviation, *IQR* interquartile range.

Over the study period, there were 35 active weather stations in Southwestern Ontario (Supplementary Fig. S1). Some weather stations were co-located, and some were only active for parts of the study. In some census divisions, there was no station or a limited number of stations. On some days, there were no temperature observations available.

**Figure 2:**
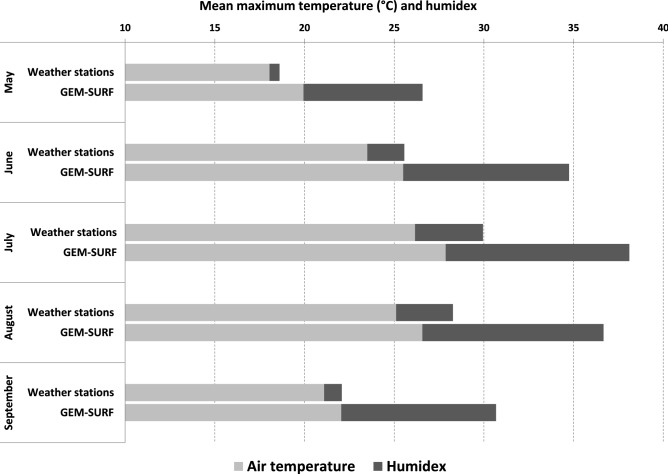
Comparison between mean maximum air temperature and humidex assigned by weather stations vs. GEM-SURF across urban Southwestern Ontario (May–September 2005–2012).

When compared with temperature data from GEM-SURF, weather stations yielded lower temperatures. Differences were most apparent with use of humidex (Fig. 2). Summaries of GEM-SURF and weather station temperatures by month and census division are presented in Supplementary Table S3.

**Figure 3:**
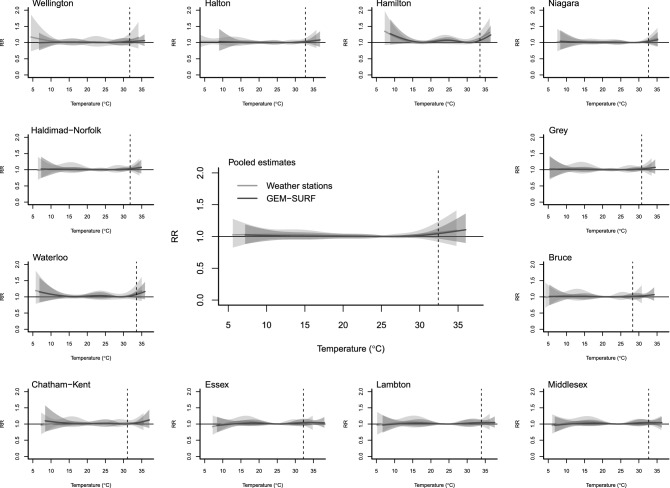
Cumulative association between mortality and maximum daily temperature by region and pooled across Southwestern Ontario between May–September 2005–2012, centered at minimum mortality temperature. Curves are presented using both GEM-SURF and weather station data. The dashed line represents the 99th percentile of weather stations temperature distribution.

Figure 3 illustrates temperature–mortality relationships using both GEM-SURF and weather stations by census division and pooled across urban Southwestern Ontario. Using GEM-SURF the MMT was 26.3 °C. There was a U-shaped association between temperature and mortality with a higher risk of mortality at lower and higher temperatures. Associations appeared slightly stronger earlier and later in the season, but overall there was no statistically significant association observed. Using weather stations, the MMT was 24.2 °C. There was also a U-shaped association between temperature and mortality, but effect estimates had slightly wider confidence intervals, especially at temperature extremes. With weather stations, there was also no association between temperature and mortality.

### Additional analyses

Increasing the number of knots and lags produced similar temperature–mortality relationships, though models had lower Quasi-Akaike information criterion values, indicating a slightly better fit. When we adjusted our main analysis for air pollution, we excluded four census divisions due to a limited number of monitoring stations; temperature–mortality relationships remained similar.

When we examined humidex-mortality relationships, the MMT was 32.6 with GEM-SURF and 28.2 for weather stations. While a U-shaped association was apparent using both data sources, at extreme humidex, the relationship was statistically significant only when weather station data was used.

In post-hoc analyses, we examined the association between mortality and extreme hot temperature [i.e. temperatures at the 99th percentile] using both data sources. There was no statistically significant relationship between temperature and mortality observed (pooled RR GEM-SURF 1.05, 95% CI 0.97–1.13; weather stations RR 1.06, 95% CI 0.92–1.23) (Table 2). Similar results were noted when we used the 75th percentile as the referent (Table 2, Fig. S3). Further, there was no significant association between minimum temperature and mortality using either MMT or the 75th percentile as the referent (Supplementary Materials Table S4, Figs. S4 and S5).

**Table 2:** Mortality risk estimates at the 99th percentile of weather station daily maximum temperature vs. the 75th percentile and vs. the minimum mortality temperature (May–September 2005–2012).

	GEM–SURF				Weather stations			
	99th vs. 75th	RR (CI)	99th vs. MMT	RR (CI)	99th vs. 75th	RR (CI)	99th vs. MMT	RR (CI)
Overall	32.4 vs. 28.1	1.04 (0.96–1.13)	32.4 vs. 26.3	1.05 (0.97–1.13)	32.4 vs. 26.6	1.06 (0.90–1.24)	32.4 vs. 24.2	1.06 (0.92–1.23)
Wellington	31.7 vs. 27.3	1.02 (0.98–1.07)	31.7 vs. 24	1.03 (0.95–1.12)	31.7 vs. 25.9	1.07 (0.95–1.20)	31.7 vs. 15.5	1.07 (0.89–1.29)
Halton	32.8 vs. 28.2	1.02 (0.98–1.07)	32.8 vs. 28.1	1.02 (0.98–1.07)	32.8 vs. 26.3	1.04 (0.93–1.16)	32.8 vs. 24.9	1.04 (0.95–1.14)
Hamilton	33.5 vs. 28.7	1.05 (0.99–1.11)	33.5 vs. 30.5	1.07 (1–1.14)	33.5 vs. 27.4	1.09 (0.99–1.21)	33.5 vs. 16.5	1.14 (0.98–1.33)
Niagara	32.6 vs. 28.3	1.03 (0.97–1.10)	32.6 vs. 28.3	1.03 (0.97–1.10)	32.6 vs. 26.9	1.06 (0.95–1.17)	32.6 vs. 28	1.06 (0.95–1.18)
Haldimand–Norfolk	31.8 vs. 27.9	1.03 (0.98–1.07)	31.8 vs. 26.8	1.03 (0.98–1.08)	31.8 vs. 26.1	1.03 (0.91–1.16)	31.8 vs. 23.7	1.04 (0.96–1.13)
Waterloo	33.6 vs. 27.7	1.07 (0.98–1.17)	33.6 vs. 29.4	1.08 (0.97–1.20)	33.6 vs. 27	1.11 (0.94–1.31)	33.6 vs. 16.1	1.12 (0.90–1.40)
Chatham–Kent	31.1 vs. 29.3	0.10 (0.97–1.03)	31.1 vs. 30.4	1 (0.99–1.01)	31.1 vs. 26.1	1.03 (0.97–1.08)	31.1 vs. 23.7	1.04 (0.96–1.12)
Essex	32.2 vs. 29.9	1.02 (0.99–1.05)	32.2 vs. 26	1.04 (0.94–1.16)	32.2 vs. 27.2	1.02 (0.93–1.12)	32.2 vs. 24.4	1.04 (0.96–1.12)
Lambton	33.9 vs. 28.7	1.03 (0.98–1.08)	33.9 vs. 24.8	1.04 (0.96–1.13)	33.9 vs. 27.1	1.03 (0.90–1.18)	33.9 vs. 24.4	1.04 (0.94–1.15)
Middlesex	32.7 vs. 28.2	1.03 (0.98–1.07)	32.7 vs. 24.4	1.04 (0.97–1.13)	32.7 vs. 27.2	1.02 (0.95–1.10)	32.7 vs. 24.1	1.04 (0.97–1.12)
Bruce	28.3 vs. 26.6	1.01 (0.97–1.04)	28.3 vs. 24.5	1.01 (0.92–1.11)	28.3 vs. 23.6	1.03 (0.92–1.15)	28.3 vs. 21.1	1.04 (0.96–1.12)
Grey	30.8 vs. 26.7	1.02 (0.98–1.06)	30.8 vs. 25.4	1.02 (0.97–1.08)	30.8 vs. 24.9	1.03 (0.92–1.16)	30.8 vs. 22.5	1.04 (0.96–1.13)

Both pooled estimate and results by region are presented.

In our exploration of MFT in our region, we found that overall, MFT was similar to MMT when GEM-SURF was used (range of absolute differences between MMT and MFT was 0.3–4.3 °C) (Supplementary Fig. S6). A slightly wider range in temperature was observed with weather stations (absolute differences, 0.5–8.3 °C), which appeared to be due to three regions (Wellington, Hamilton, and Waterloo) where there was a lower MMT than MFT. When MFT was used as our referent rather than MMT, we did not observe any change to temperature–mortality relationships (Supplementary Fig. S7).

## Discussion

In this large population-based study of temperature–mortality relationships in urban Southwestern Ontario, we found that higher temperatures were consistently captured by GEM-SURF, particularly with use of humidex. Use of GEM-SURF also produced temperature–mortality effect estimates with narrower confidence intervals. These findings may have been due to more complete temperature coverage with GEM-SURF, and the relative scarcity of weather stations. Weather stations are also typically located near airports, water or over grassy areas in non-urban regions and might not capture the temperature exposure of urban inhabitants well^10,36^. This is in contrast with GEM-SURF which ascertains temperature across all surfaces including paved areas, and can validly capture the urban heat island effect.

Despite differences in assigned temperatures using different data sources, we found that both produced similar maximum and minimum daily temperature–mortality relationships. Results remained robust in additional analyses which included use of different referent temperatures. Although previous studies have not used validated urban temperature sources like GEM-SURF, our findings are consistent with Guo et al. (2000–2004) who found that use of interpolated temperatures in Brisbane City Australia, produced a wider range of temperatures than weather stations, but similar temperature–mortality relationships^45^. In Paris (2000–2006), Schaeffer et al. examined use of single, average and population weighted averages of temperature across weather stations, and population-weighted averages of temperature from classifications based upon land use. They also noted similar temperature–mortality relationships using all temperature data sources^38^.

Our results differ from authors who observed different temperature–mortality/morbidity relationships when different temperature data sources were used. Weinberger et al. examined the relationship between temperature and mortality using the Parameter elevation Regression on Independent Slopes Model (PRISM) vs. weather stations across the United States. In the majority of counties, PRISM led to slightly larger relative risks of death with high temperature compared with weather stations^14^. Lee et al. examined the association between modelled daily mean air temperature (1 km resolution aggregated to the zip code level) and non-accidental mortality in urban and rural regions of the Carolina and Georgia states (2007–2011). There was a 2.05% (0.87–3.24%) increase in mortality for each 1° increase in temperature above 28 °C, with an effect size that was 79.8% higher with modelled data^11^. Although their focus was on morbidity, Adeyeye et al. examined the relationship between maximum near-surface temperature and morbidity (i.e. heat stress, dehydration) using a gridded National Land Data Assimilation System and weather stations between 2008 and 2012 in New York State. There was an increased risk of all health outcomes with high temperature, and risk estimates were attenuated with wider confidence intervals when weather stations were used^12^. Finally, in a Veteran’s Affairs study of men in the Normative Aging study, Zanobetti et al. found that for every 1 °C increase in temperature, the odds of having ectopy was 1.10 (95% CI 1.04–1.17). The magnitude was attenuated when weather station data was used^46^.

Reasons for observed differences between our studies and the latter studies might be manifold. Studies were conducted outside of Canada or not focused on cities, and there are known area-level differences in temperature susceptibility due to geographic, built environment, demographic and the social characteristics of populations^14,47–50^. Studies used different methodologies (i.e. case-crossover logistic regression), as well as different data sources which did not include urban modelling.

Of note, other Canadian studies have observed a positive association between temperature/temperature variability and mortality when weather stations were used^2,51,52^. Studies were however, conducted outside of Ontario, or were inclusive of Toronto Ontario (Ontario’s largest city, excluded from our study)^51,52^. Interestingly, one study was a 15-year investigation of temperature–mortality relationships across 49 census divisions in Ontario (1996–2010). Authors observed a 2.5% increase in mortality (95% CI 1.3–3.8%) per 5 °C increase in daily mean temperature during warm seasons. However, estimates for census divisions in Southwestern Ontario (our region of study) were not statistically significant, or only marginally significant^2^. It also remains possible that the null relationship we observed, was due to limited statistical power as some of the regions included had few deaths over the study period (Supplementary Table S3).

We conducted a large, Canadian, population-based time series analysis using high quality administrative health and environmental datasets. We focused upon an understudied region of our province, urban Southwestern Ontario. We explored two temperature data sources available to environmental health researchers, highlighted their strengths and limitations, and contrasted temperature–mortality relationships with use of both datasets. We also used a validated urban data source (GEM-SURF) which can accurately capture urban-scale climate. Further, we conducted numerous pre-specified and post-hoc additional analyses to enrich our results which included an examination of extreme minimum temperature–mortality relationships, and an investigation of temperature–mortality relationships using different referent temperatures.

GEM-SURF is a numerical weather prediction model that requires multiple inputs and thus, there could be uncertainties associated with model formulation. Another limitation of our study was that we only had access to GEM-SURF data to the end of 2012, and cannot extrapolate results to more recent years. The highest resolution we could aggregate temperature to was the DA level, and we may have lost data granularity. However in urban areas, DAs are very small regions and we likely maintained spatial variability and urban effects. Additionally, we used exposure data from sparsely distributed weather stations across the province, many of which were located in coastal areas. Use of these stations might have captured lower temperatures in the summer due to daytime lake-breeze effects.

As with most environmental health studies, we also could not confirm true heat exposure (e.g. residents may have been indoors at the time of death). Further, temperature exposure was assigned at location of residence and we could not account for dynamic populations (e.g. travel outside of residential location)^6,12^. Although we were able to characterize some of the characteristics of individuals who died, we could not capture factors including poor housing quality, air conditioning, access to care, or individual behaviors that might have modified associations^53^. This was also a population-level study and we cannot assume that the relationship between temperature and mortality holds at an individual level. Finally, this work is only generalizable to urban Southwestern Ontario.

## Conclusion

Use of GEM-SURF in urban environmental health studies might be desirable where station observations do not fully capture population exposure (e.g. population lives distant from stations). GEM-SURF might also be ideal where capturing the magnitude of temperature exposure at a local level is particularly important. However, if focused upon the relationship between mortality and maximum or minimum temperature using time series in large urban regions, either temperature data source appears to produce similar temperature–mortality associations.

## Supplementary Information

Supplementary Information.

## Data Availability

The dataset from this study is held securely in coded form at ICES. While data sharing agreements prohibit ICES from making the dataset publicly available, access may be granted to those who meet pre-specified criteria for confidential access, available at www.ices.on.ca/DAS. The full dataset creation plan and underlying analytic code are available from the authors upon request, understanding that the computer programs may rely upon coding templates or macros that are unique to ICES and are therefore either inaccessible or may require modification.
