# Supplementary material for: Evaluating the association between extreme heat and mortality in urban Southwestern Ontario using different temperature data sources
Source: Sci Rep. 2021 Apr 14;11:8153. doi: 10.1038/s41598-021-87203-0 (PMC8046761; doi:10.1038/s41598-021-87203-0)
Supplement: Supplementary file 1 — Supplementary Information. [file 41598_2021_87203_MOESM1_ESM.docx]

**Supplementary Information**

**Evaluating the association between extreme heat and mortality in urban Southwestern Ontario using different temperature data sources**

Kristin K. Clemens MD MSc; Alexandra M. Ouédraogo MSc; Lihua Li PhD; James A. Voogt PhD; Jason Gilliland PhD; E. Scott Krayenhoff PhD; Sylvie Leroyer PhD; Salimah Z. Shariff PhD

Corresponding Author: Kristin K. Clemens, Western University, Email: kristin.clemens@sjhc.london.on.ca

**Table of Contents**

**Table S1.** RECORD checklist of recommendations for the reporting of studies conducted using routinely collected health data

**Figure S1.** Location of active weather stations (n=35) and air quality stations (n=16) in Southwestern Ontario between 2005–2012

**Table S2.** Coding definitions of study variables

**Figure S2.** Flow diagram of study inclusions and exclusions

**Table S3.** Summary of mortality and temperature exposure by subregion, May–September 2005–2012

**Table S4.** Mortality risk estimates at the 99th percentile of weather station daily minimum temperature, compared to the 75^th^ percentile and vs. minimum mortality temperature (May–September 2005–2012). Both pooled estimate and results by region are presented.

**Figure S3.** Cumulative association between mortality and maximum daily temperature across all regions in Southwestern Ontario between May–September 2005–2012, centered at 75^th^ percentile of temperature. Curves are presented using both GEM–SURF and weather station data. The dashed line represents the 99^th^ percentile of weather stations temperature distribution.

**Figure S4.** Cumulative association between mortality and minimum daily temperature across all regions in Southwestern Ontario between May–September 2005–2012, centered at 75^th^ percentile of temperature. Curves are presented using both GEM–SURF and weather station data. The dashed line represents the 99^th^ percentile of weather stations temperature distribution

**Figure S5.** Cumulative association between mortality and minimum daily temperature across all regions in Southwestern Ontario between May–September 2005–2012, centered at minimum mortality temperature. Curves are presented using both GEM–SURF and weather station data. The dashed line represents the 99^th^ percentile of weather stations temperature distribution.

**Figure S6.** Comparison between minimum mortality temperature and most frequent temperature (MFT). MFT was calculated using the mode of daily maximum temperature distributed within the using the 95% distribution of minimum mortality percentiles(i.e. 56^th^ –72^nd^ range for GEM–SURF, and 32^nd^–58^th^ range for weather stations)

**Figure S7.** Cumulative association between mortality and maximum daily temperature, pooled across all regions in Southwestern Ontario between May–September 2005–2012, centered at the MFT. Curves are presented using both GEM–SURF and weather station data. The dashed line represents the 99^th^ percentile of weather stations temperature distribution.

**Table S1. RECORD checklist of recommendations for the reporting of studies conducted using routinely collected health data**

|  | **Item No** | **Recommendation** | **Reported** |
| --- | --- | --- | --- |
| Title and abstract | 1 | 1.1 The type of data used should be specified in the title or abstract. When possible, the name of the databases should be included. | Abstract page 2 |
|  |  | 1.2 If applicable, the geographic region and time frame within which the study took place should be reported in the title or abstract. | Abstract page 2 |
|  |  | 1.3 If linkage between databases was conducted for the study, this should be clearly stated in the title or abstract | Abstract page 2 |
| Introduction | | |  |
| Background/rationale | 2 | Explain the scientific background and rationale for the investigation being reported | Background page 3-4 |
| Objectives | 3 | State specific objectives, including any pre–specified hypotheses | Background page 4 |
| Methods | | |  |
| Study design | 4 | Present key elements of study design early in the paper | Methods page 4-5 |
| Setting | 5 | Describe the setting, locations, and relevant dates, including periods of recruitment, exposure, follow–up, and data collection | Methods page 4-5 |
| Participants | 6 | 6.1 The methods of study population selection should be listed in detail. If this is not possible, an explanation should be provided. | Methods page 5 |
|  |  | 6.2 Any validation studies of the codes or algorithms used to select the population should be referenced. If validation was conducted for this study and not published elsewhere, detailed methods and results should be provided. | NA |
|  |  | 6.3 If the study involved linkage of databases, consider use of a flow diagram or other graphical display to demonstrate the linkage process, including the number of individuals with linked data at each stage. | Supplementary Figure S3 |
| Variables | 7 | A complete list of codes and algorithms used to classify exposures, outcomes, confounders, and effect modifiers should be provided. If these cannot be reported, an explanation should be provided. | Supplementary Table S2 |
| Data sources/ measurement | 8 | For each variable of interest, give sources of data and details of methods of assessment (measurement). Describe comparability of assessment methods if there is more than one group | Supplementary Table S2 |
| Bias | 9 | Describe any efforts to address potential sources of bias | Methods page 9-10 |
| Study size | 10 | Explain how the study size was arrived at | Supplementary Figure S3 |
| Quantitative variables | 11 | Explain how quantitative variables were handled in the analyses. If applicable, describe which groupings were chosen and why | Methods page 8-9, Table 1 |
| Statistical methods | 12 | 12.1 Describe all statistical methods, including those used to control for confounding | Methods page 8–10 |
|  |  | 12.2 Describe any methods used to examine subgroups and interactions | Methods page 8–10 |
|  |  | 12.3 Explain how missing data were addressed | Methods page 8–10 |
|  |  | 12.4 If applicable, explain how loss to follow–up was addressed | NA |
|  |  | 12.5 Describe any sensitivity analyses | Methods page 9–10 |
| Data access and cleaning methods |  | 12.6 Authors should describe the extent to which the investigators had access to the database population used to create the study population. | Methods page 5 |
|  |  | 12.7 Authors should provide information on the data cleaning methods used in the study | Methods page 5-6 |
| Linkage |  | 12.8 State whether the study included person–level, institutional–level, or other data linkage across two or more databases. The methods of linkage and methods of linkage quality evaluation should be provided. | Methods page 5–6 |
| Results | | |  |
| Participants | 13 | 13.1 Describe in detail the selection of the persons included in the study (i.e. study population selection), including filtering based on data quality, data availability, and linkage. The selection of included persons can be described in the text and/or by means of the study flow diagram. | Supplementary Figure S3 |
| Descriptive data | 14 | 14.1 Give characteristics of study participants (e.g. demographic, clinical, social) and information on exposures and potential confounders | Results page 10, Table 1, Supplementary Table S3 |
|  |  | 14.2 Indicate number of participants with missing data for each variable of interest | Table 1 |
|  |  | 14.3 Summarize follow–up time (e.g. average and total amount) | NA |
| Outcome data | 15 | Report numbers of outcome events or summary measures over time | Table S3 |
| Main results | 16 | 16.1 Give unadjusted estimates and, if applicable, confounder–adjusted estimates and their precision (e.g. 95% confidence interval). Make clear which confounders were adjusted for and why they were included | Results page 10–12, Table 2, Figure 3, Table S4, Figure S3-S7 |
|  |  | 16.2 Report category boundaries when continuous variables were categorized | Table 1 |
|  |  | 16.3 If relevant, consider translating estimates of relative risk into absolute risk for a meaningful time period | NA |
| Other analyses | 17 | Report other analyses done–e.g. analyses of subgroups and interactions, and sensitivity analyses | Results page 11–12 |
| Discussion | | |  |
| Key results | 18 | Summarize key results with reference to study objectives | Discussion page 12-13 |
| Limitations | 19 | Discuss the implications of using data that were not created or collected to answer the specific research question(s). Include discussion of misclassification bias, unmeasured confounding, missing data and changing eligibility over time, as they pertain to the study being reported. | Discussion page 13–16 |
| Interpretation | 20 | Give a cautious overall interpretation of results considering objectives, limitations, multiplicity of analyses, results from similar studies, and other relevant evidence | Discussion page 13–16 |
| Generalizability | 21 | Discuss the generalizability (external validity) of the study results | Discussion page 15-16 |
| Other information | | |  |
| Funding | 22 | 22.1 Give the source of funding and the role of the funders for the present study and, if applicable, for the original study on which the present article is based | Funding |
| Accessibility of protocol, raw data and programming code |  | 22.2 Authors should provide information on how to access any supplemental information such as the study protocol, raw data, or programming code. | The dataset from this study is held securely in coded form at ICES. While data sharing agreements prohibit ICES from making the dataset publicly available, access may be granted to those who meet pre–specified criteria for confidential access, available at [www.ices.on.ca/DAS](http://www.ices.on.ca/DAS). The full dataset creation plan and underlying analytic code are available from the authors upon request, understanding that the computer programs may rely upon coding templates or macros that are unique to ICES and are therefore either inaccessible or may require modification |

**Figure S1. Location of active weather stations (n=35) and air quality stations (n=16) in Southwestern Ontario between 2005–2012.**

**
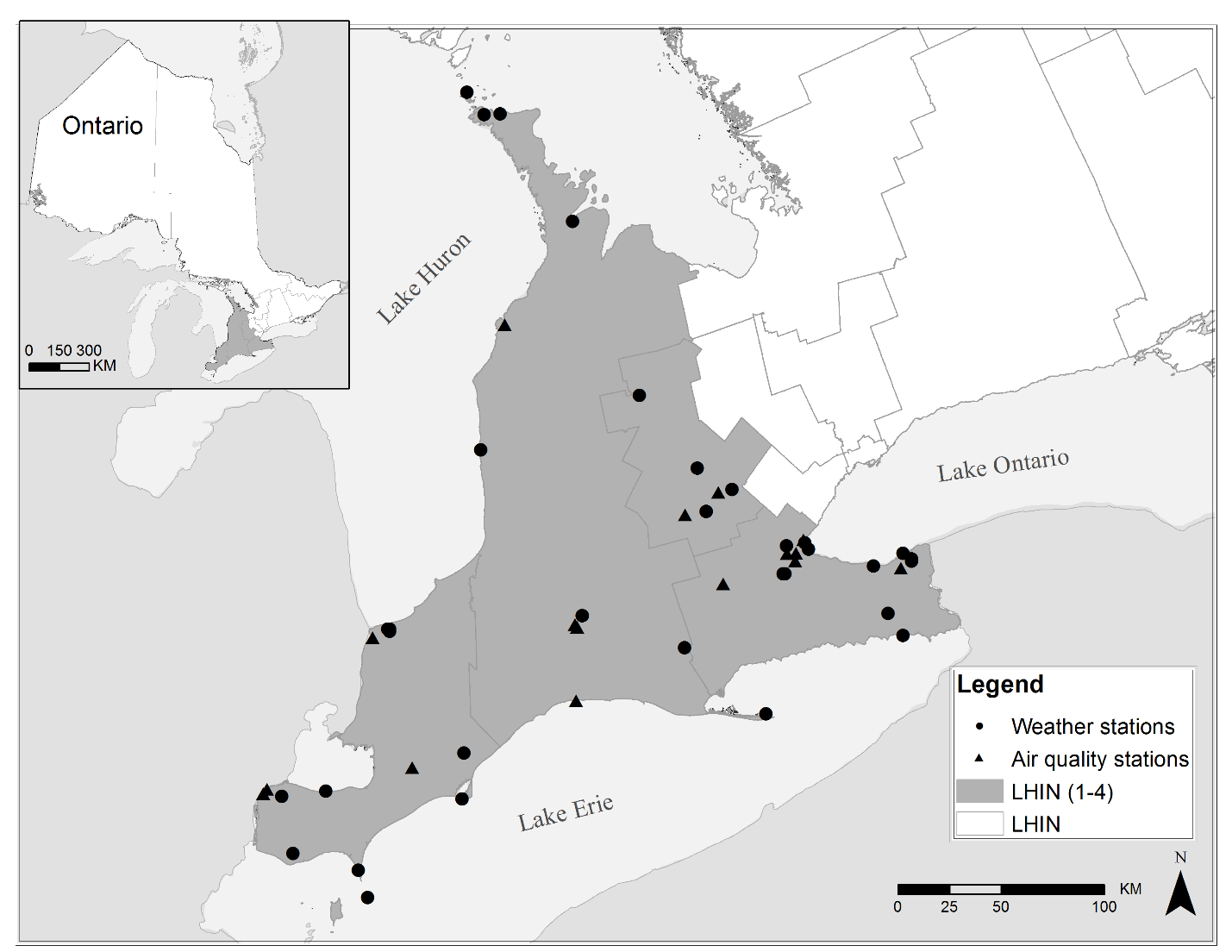
**

There were 35 unique weather stations that were active at some point over the study period. There are 29 stations presented here as some were co–located or were only active during a short period of time. Map created by A.O using ArcGIS software version 10.3 by ESRI, [www.esri.com](http://www.esri.com) .

**Table S2. Coding definitions of study variables**

| **Variable** | **Database** | **Codes** |
| --- | --- | --- |
| Age | RPDB |  |
| Female | RPDB |  |
| LHIN | RPDB  LHIN |  |
| Neighbourhood income quintile | RPDB |  |
| Marginalization index | ON MARG |  |
| Congestive heart failure | CHF | Diagnosis in CHF database as of the index date |
| Chronic obstructive pulmonary disease | COPD | Diagnosis in COPD database as of the index date |
| Hypertension | HYPER | Diagnosis in HYPER database as of the index date |
| Diabetes | ODD | Diagnosis in ODD database as of the index date |
| Coronary artery disease (excluding angina) | CIHI–DAD  NACRS  OHIP | ICD 9: "412", "410", "411"  ICD 10: "I21", "I22", "Z955", "T822"  CCP: "4801", "4802", "4803", "4804", "4805", "481", "482", "483"  CCI: "1IJ50", "1IJ76"  OHIP Fee: "R741", "R742", "R743", "G298", "E646", "E651", "E652", "E654", "E655", "Z434", "Z448"  OHIP Dx: "410", "412" |
| Cerebrovascular disease | CIHI–DAD | ICD 9: "430", "431", "432", "434", "435", "436", "3623"  ICD 10: "I62", "I630", "I631", "I632", "I633", "I634", "I635", "I638", "I639", "I64", "H341", "I600", "I601", "I602", "I603", "I604", "I605", "I606", "I607", "I609", "I61", "G450", "G451", "G452", "G453", "G458", "G459", "H340" |
| Dementia | CIHI–DAD  NACRS  OHIP | ICD 9:"2900", "2901", "2902", "2903", "2904", "2908", "2909", "2948", "2949", "3310", "3311", "3312", "2941", "797"  ICD 10: "F065", "F066", "F068", "F069", "F09", "F00", "F01", "F02", "F03", "F051", "G30", "G31", "R54"  OHIP DX: "290","331", "797" |
| Chronic kidney disease | CIHI–DAD  NACRS  OHIP | ICD 9: "4030", "4031", "4039", "4040", "4041", "4049", "583", "584", "585", "586", "5888", "5889", "592", "5939", "2504"  ICD 10: "E102", "E112", "E132", "E142", "I12", "I13", "N00", "N01", "N02", "N03", "N04", "N05", "N06", "N07", "N08", "N10", "N11", "N12", "N13", "N14", "N15", "N16", "N17", "N18", "N19", "N20", "N21", "N22", "N23"  OHIP DX: "403", "585" |

Abbreviations: CCI, Canadian Classification of Health Interventions; CCP, Canadian Classification of Diagnostic, Therapeutic and Surgical Procedures; CIHI–DAD, Canadian Institute for Health Information’s Discharge Abstract Database; Dx, Diagnostic; ICD, International Classification of Diseases; NACRS, National Ambulatory Care Reporting System Database; ODD, Ontario Diabetes Database; OHIP, Ontario Health Insurance Plan; RPDB, Registered Persons Database

**Figure S2. Flow diagram of study inclusion and exclusions**

Exclusions:
Invalid identification number n=0

Missing or invalid age n=71

Missing sex n=0

Not a permanent resident of Ontario n=30

Does not reside in an urban area n=33,996

Lives in long–term care n=51,711

Accidental cause of death n=13,466

Ontario residents living in LHIN 1, 2, 3 or 4 who died between Jan 1, 2005–December 31, 2012

N=236,111

Died from May 1–September 30 2005–2012

N=54,399

Remaining cohort

N=136,837

**Table S3. Summary of mortality and temperature exposure by subregion, May-Sept 2005-2012**

| **Locations (Census Divisions)** | **Total number of deaths** | **Maximum temperature (°C) (source: GEM-SURF)** | **Maximum temperature (°C) (source: weather stations)** | **Maximum humidex (°C) (source: GEM-SURF)** | **Maximum humidex (°C) (source: weather stations)** |
| --- | --- | --- | --- | --- | --- |
| Wellington | 2,462 | 24.0 (6.3–35.8) | 22.8 (4.5–34.1) | 32.8 (9.2–51.3) | 22.8 (4.5–44.3) |
| Halton | 2,767 | 24.7 (8.9–36.8) | 22.5 (6.6–35.8) | 33.9 (11.5–53.2) | 22.5 (6.6–43.8) |
| Hamilton | 9,138 | 25.5 (8.7–36.6) | 24.1 (7.3–36.2) | 34.2 (11.3–53.3) | 25.3 (7.3–49.1) |
| Niagara | 9,051 | 25.2 (8.2–35.2) | 23.8 (7.5–35.2) | 34.28 (11.0–51.5) | 23.9 (7.5–45.7) |
| Haldimand–Norfolk | 1,553 | 24.8 (7.3–35.0) | 23.4 (6.4–34.4) | 34.3 (10.8–52.1) | 23.8 (6.4–47.0) |
| Brant* | 2,341 | 25.2 (7.9–36.5) | NA | 34.2 (10.5–53.0) | NA |
| Waterloo | 6,249 | 24.4 (6.7–36.0) | 24.0 (5.8–35.0) | 33.0 (9.1–51.0) | 24.0 (5.8–46.4) |
| Perth* | 633 | 24.0 (5.8–36.0) | NA | 33.0 (8.4–52.5) | NA |
| Oxford* | 1,302 | 24.7 (6.9–36.1) | NA | 33.8 (9.5–52.6) | NA |
| Elgin* | 1,312 | 24.8 (6.2–35.6) | NA | 34.3 (10.2–51.6) | NA |
| Chatham–Kent | 2,252 | 26.1 (8.4–37.2) | 23.3 (7.3–35.5) | 35.6 (12.2–54.0) | 23.7 (7.3–46.9) |
| Essex | 6,155 | 26.6 (8.4–38.1) | 24.4 (7.2–34.8) | 35.8 (12.2–54.0) | 25.8 (7.2–49.1) |
| Lambton | 1,835 | 25.2 (6.6–37.7) | 23.8 (5.2–36.3) | 34.5 (10.9–53.4) | 23.8 (5.2–47.6) |
| Middlesex | 6,542 | 25.1 (6.8–36.3) | 24.2 (6.1–36.2) | 34.0 (9.2–53.2) | 24.2 (6.1–45.9) |
| Bruce | 55 | 23.5 (5.2–34.3) | 21.0 (4.2–30.8) | 32.4 (7.8–50.7) | 21.0 (4.2–39.2) |
| Grey | 752 | 23.4 (5.4–34.5) | 21.9 (5.4–33.5) | 32.2 (7.9–49.9) | 21.9 (5.4–43.6) |

* Locations were excluded from weather stations analysis, as there were no stations that fell within these census divisions.

**Table S4. Mortality risk estimates at the 99th percentile of weather station daily minimum temperature vs the 75^th^ percentile and vs the minimum mortality temperature (May–September 2005–2012). Both pooled estimate and results by region are presented.**

|  | **GEM–SURF** | | | | **Weather stations** | | | |
| --- | --- | --- | --- | --- | --- | --- | --- | --- |
| **Locations** | **99^th^ vs. 75^th^** | **RR (CI)** | **99^th^ vs. MMT** | **RR (CI)** | **99^th^ vs. 75^th^** | **RR(CI)** | **99^th^ MMT** | **RR (CI)** |
| **Overall** | **22.2 vs. 17.8** | **1.028 (0.973-1.086)** | **22.2 vs. 15.9** | **1.03 (0.973–1.09)** | **22.2 vs. 16.9** | **1.099 (0.971-1.244)** | **22.2 vs. 15.9** | **1.101 (0.97–1.249)** |
| Wellington | 20.6 vs. 16.7 | 1.027 (0.981-1.074) | 20.6 vs. 12.8 | 1.047 (0.939–1.167) | 20.6 vs. 14.6 | 1.113 (0.995-1.246) | 20.6 vs. 9.9 | 1.134 (0.982–1.309) |
| Halton | 23.9 vs. 18.2 | 1.039 (0.966-1.118) | 23.9 vs. 19.1 | 1.041 (0.962–1.126) | 23.9 vs. 18.9 | 1.065 (0.953-1.19) | 23.9 vs. 20.1 | 1.07 (0.967–1.185) |
| Hamilton | 22 vs. 18.5 | 1.001 (0.961-1.044) | 22.1 vs. 20.3 | 1.012 (0.989–1.036) | 22 vs. 16.3 | 1.054 (0.956-1.161) | 22.1 vs. 17.9 | 1.061 (0.972–1.157) |
| Niagara | 23.5 vs. 18.7 | 1.044 (0.97-1.124) | 23.5 vs. 20.3 | 1.054 (0.975–1.14) | 23.5 vs. 18.5 | 1.051 (0.949-1.163) | 23.5 vs. 20.2 | 1.063 (0.972–1.162) |
| Haldimand–Norfolk | 22.3 vs. 17.4 | 1.032 (0.971-1.097) | 22.3 vs. 13.7 | 1.049 (0.953–1.155) | 22.3 vs. 17.5 | 1.077 (0.972-1.194) | 22.3 vs. 17.3 | 1.077 (0.97–1.196) |
| Waterloo | 21.6 vs. 17.4 | 1.023 (0.975-1.075) | 21.6 vs. 15.9 | 1.025 (0.959–1.095) | 21.6 vs. 15 | 1.084 (0.975-1.206) | 21.6 vs. 6 | 1.088 (0.948–1.25) |
| Chatham–Kent | 23.1 vs. 18.5 | 1.025 (0.973-1.079) | 23.1 vs. 19 | 1.025 (0.977–1.075) | 23.1 vs. 17.9 | 1.073 (0.958-1.201) | 23.1 vs. 18.7 | 1.075 (0.967–1.196) |
| Essex | 23.6 vs. 19.1 | 1.029 (0.978-1.082) | 23.6 vs. 15.8 | 1.039 (0.949–1.138) | 23.6 vs. 18.8 | 1.062 (0.97-1.163) | 23.6 vs. 19.1 | 1.062 (0.973–1.16) |
| Lambton | 22.3 vs. 18.2 | 1.016 (0.968-1.066) | 22.3 vs. 18.3 | 1.016 (0.97–1.065) | 22.3 vs. 17.3 | 1.055 (0.964-1.153) | 22.3 vs. 17.8 | 1.055 (0.971–1.147) |
| Middlesex | 21.8 vs. 18 | 1.03 (0.985-1.077) | 21.8 vs. 14.4 | 1.052 (0.956–1.156) | 21.8 vs. 16.5 | 1.061 (0.974-1.156) | 21.8 vs. 16.1 | 1.061 (0.97–1.161) |
| Bruce | 20.2 vs. 17.1 | 1.014 (0.975-1.055) | 20.2 vs. 14.7 | 1.018 (0.924–1.121) | 20.2 vs. 15.1 | 1.088 (0.966-1.226) | 20.2 vs. 15.1 | 1.088 (0.966–1.226) |
| Grey | 20.9 vs. 16.9 | 1.015 (0.97-1.062) | 20.9 vs. 17.5 | 1.015 (0.979–1.053) | 20.9 vs. 15.2 | 1.061 (0.96-1.173) | 20.9 vs. 16 | 1.062 (0.969–1.164) |

**Figure S3. Cumulative association between mortality and maximum daily temperature across all regions in Southwestern Ontario between May–September 2005–2012, centered at 75^th^ percentile of temperature. Curves are presented using both GEM–SURF and weather station data. The dashed line represents the 99^th^ percentile of weather stations temperature distribution.**


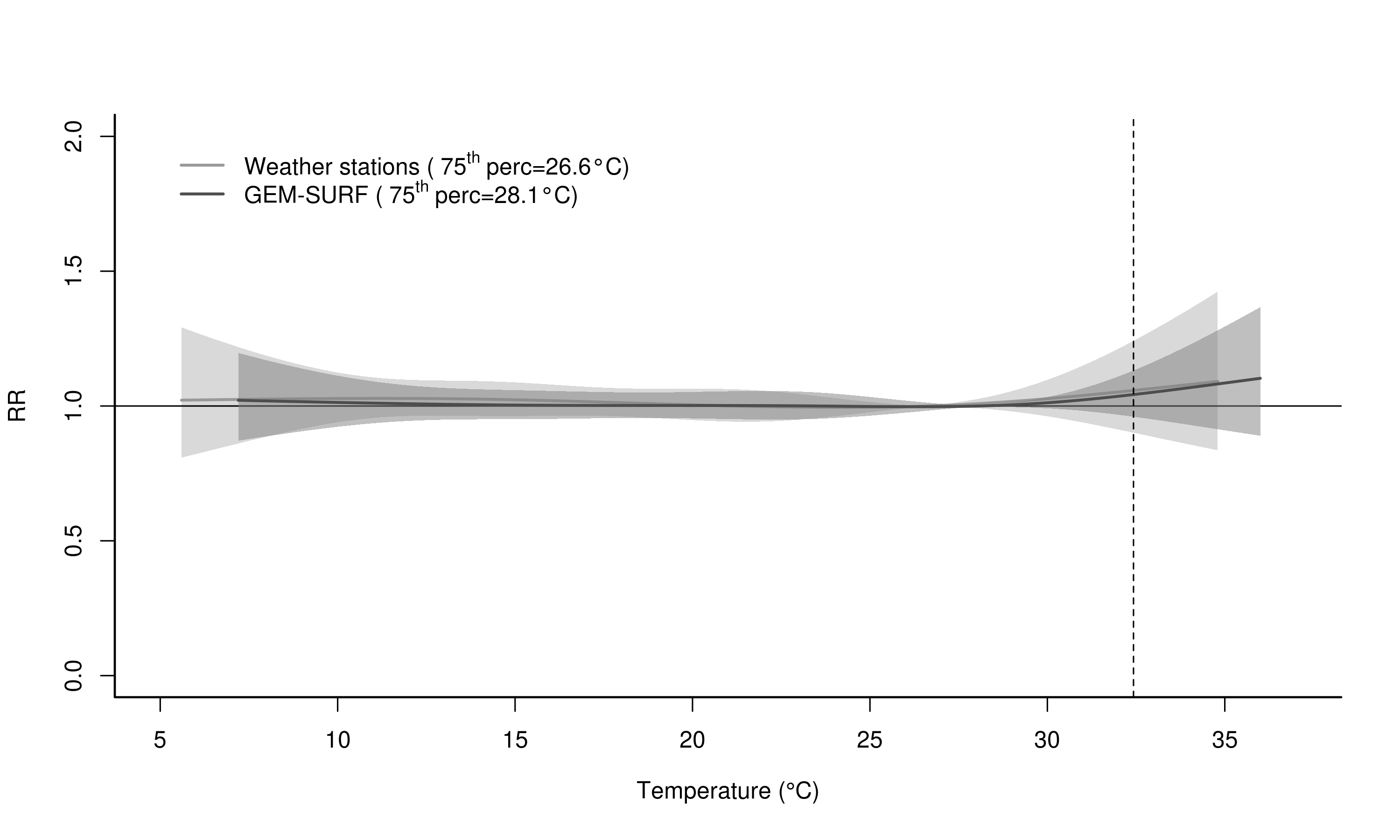


**Figure S4. Cumulative association between mortality and minimum daily temperature across all regions in Southwestern Ontario between May–September 2005–2012, centered at 75^th^ percentile of temperature. Curves are presented using both GEM–SURF and weather station data. The dashed line represents the 99^th^ percentile of weather stations temperature distribution.**


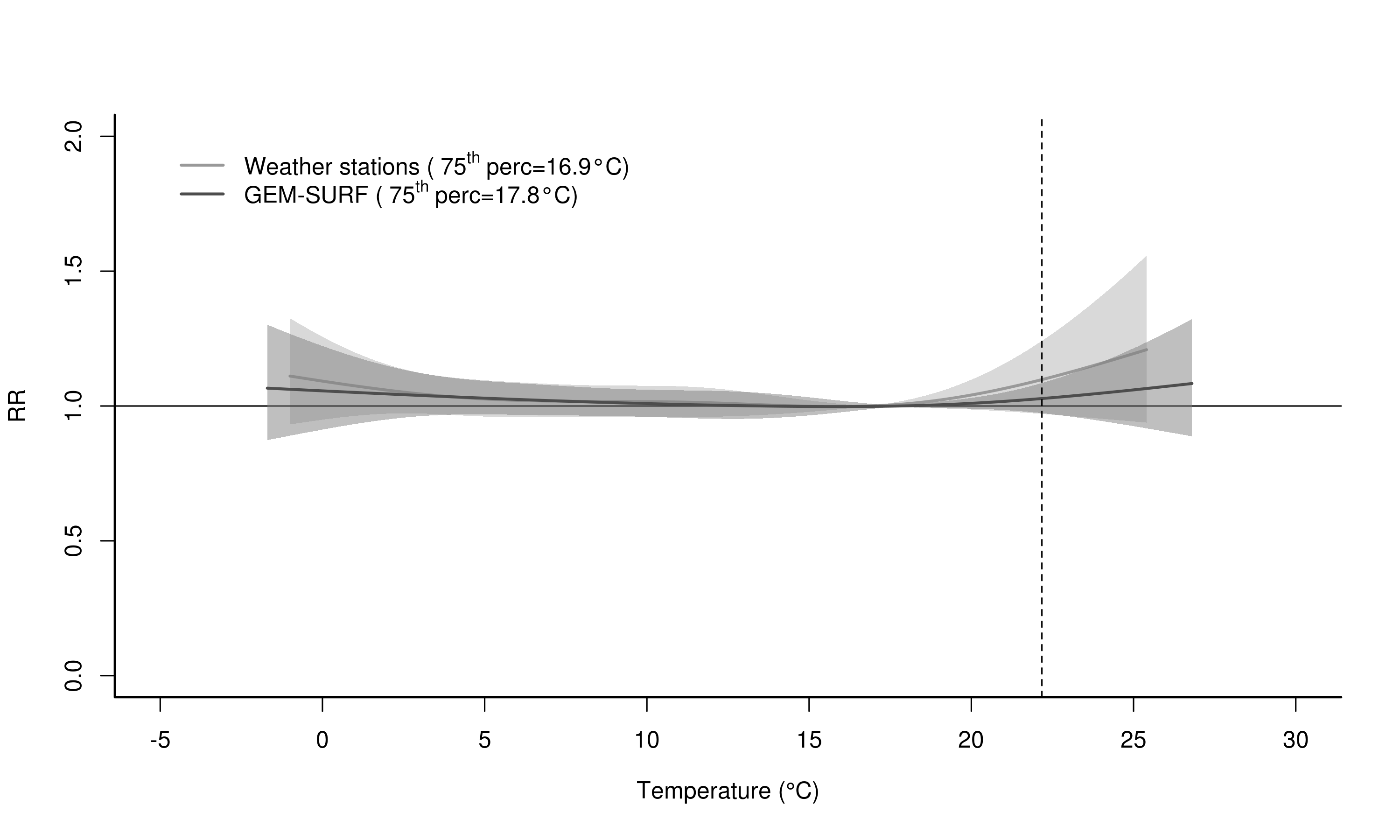


**Figure S5. Cumulative association between mortality and minimum daily temperature across all regions in Southwestern Ontario between May–September 2005–2012, centered at minimum mortality temperature. Curves are presented using both GEM–SURF and weather station data. The dashed line represents the 99^th^ percentile of weather stations temperature distribution.**

**Figure S6. Comparison between minimum mortality temperature and most frequent temperature (MFT). MFT was calculated using the mode of daily maximum temperature distributed within the 95% distribution of minimum mortality percentiles (i.e. 56^th^–72^nd^ range for GEM**–**SURF, and 32^nd^–58^th^ range for weather stations)**

**Figure S7. Cumulative association between mortality and maximum daily temperature, pooled across all regions in Southwestern Ontario between May**–**September 2005–2012, centered at the MFT. Curves are presented using both GEM–SURF and weather station data. The dashed line represents the 99^th^ percentile of weather stations temperature distribution.**
